# Higher CSF Levels of NLRP3 Inflammasome Is Associated With Poor Prognosis of Anti-N-methyl-D-Aspartate Receptor Encephalitis

**DOI:** 10.3389/fimmu.2019.00905

**Published:** 2019-05-31

**Authors:** Yu Peng, Baozhu Liu, Shanshan Pei, Dong Zheng, Zhanhang Wang, Teng Ji, Suyue Pan, Hai-Ying Shen, Honghao Wang

**Affiliations:** ^1^Department of Neurology, Nanfang Hospital, Southern Medical University, Guangzhou, China; ^2^Department of Neurology, The Affiliated Brain Hospital of Guangzhou Medical University, Guangzhou, China; ^3^Department of Neurology, Guangdong Brain Hospital, Guangzhou, China; ^4^Department of Pediatric Neurology, Legacy Emanuel Medical Center, Randall Children's Hospital, Portland, OR, United States; ^5^RS Dow Neurobiology Laboratories, Legacy Research Institute, Portland, OR, United States

**Keywords:** anti-NMDAR encephalitis, neuro-inflammation, cytokine, NLRP3, modified Rankin Scale

## Abstract

Anti-N-methyl-D-aspartate receptor (NMDAR) encephalitis is accepted as an autoimmune disorder of the central nervous system (CNS). NLR family pyrin domain containing 3 (NLRP3) inflammasome, a potent innate inflammatory mediator, can activate IL-1β and induce the migration of T helper cell into CNS. However, the possible role of NLRP3 inflammasome in the pathogenesis of anti-NMDAR encephalitis remains unclear. This study aims to determine the levels of NLRP3 and related cytokines (IL-1β, IL-6, and IL-17) in the cerebrospinal fluid (CSF) of anti-NMDAR encephalitis patients and to assess whether NLRP3 influences the clinical outcomes of this disease. Twenty-five patients with anti-NMDAR encephalitis, 12 viral meningoencephalitis patients and 26 controls with non-inflammatory neurological diseases were recruited. CSF NLRP3 inflammasome, IL-1β, IL-6, and IL-17 were measured by enzyme-linked immunosorbent assay. Thirteen out of 25 patients were re-examed for the concentrations of NLRP3 and cytokines 6 months later. Our results showed significant increases of CSF NLRP3 inflammasome, IL-1β, IL-6, and IL-17 in anti-NMDAR encephalitis patients. There were positive correlations between CSF NLRP3 inflammasome and cytokines in anti-NMDAR encephalitis patients. There was also a positive correlation between maximum modified Rankin Scale (mRS) scores and CSF NLRP3 inflammasome in anti-NMDAR encephalitis patients. During follow-up, the decrease of mRS was positively correlated with the decrease of CSF NLRP3 inflammasomes. These results suggested that the level of CSF NLRP3 inflammasome could represent the severity of anti-NMDAR encephalitis and the reduction of CSF NLRP3 inflammasome could act as an indicator for the prognosis of this disease.

## Introduction

As an autoimmune disorder, anti-N-methyl-D-aspartate receptor (NMDAR) encephalitis predominantly affects young females (1). Its incidence is approximated at 5–10 per 100,000 people per year (2, 3). The typical clinical manifestation presents as psychiatric and neurologic symptoms, such as seizures, movements disorders, autonomic dysfunction, and cognition dysfunction (4, 5). Anti-NMDAR encephalitis may be associated with teratoma and partly secondary to central nervous system (CNS) infections such as herpes simplex virus or parasite (6, 7). Although B and T leukocytes have been proposed to be involved in anti-NMDAR encephalitis, the immunopathogenesis of this disease remains obscure. We have reported several inflammatory cytokines were increased in serum or cerebrospinal fluid (CSF) of anti-NMDAR encephalitis (8–10).

The inflammasomes are cytosolic multi-protein complexes that initiate the activation of caspases-1 and subsequently the cleavage of pro-inflammatory cytokines interleukin (IL)-1β and IL-18 (11). Four different receptors have been shown to form inflammasomes, three of which, NLRP1, NLRP3, and NLRC4, belong to the Nod-like receptor (NLR) family of proteins. The fourth one, AIM2, belongs to the hematopoietic interferon-inducible nuclear (HIN) protein family (12). NLRP3 inflammasome is the most extensively studied but also the most elusive one. In response to a number of physical and chemical triggers, such as bacterial infection, extracellular ATP, glucose, and cholesterin, NLRP3 activates innate immune system and leads to tissue damage (13). Some studies have reported that NLRP3 inflammasome is of great important in the pathogenesis of some central nervous disease such as stroke, epilepsy, Alzheimer's disease (AD), and Parkinson's disease (PD) (14–16). But CSF levels of NLRP3 inflammasome in anti-NMDAR encephalitis patients has not been determined.

Previous experiments have confirmed that T helper (Th) cells might support B cell function (17, 18) and that CSF IL-6 and IL-17 were elevated in anti-NMDAR encephalitis patients (19, 20). As neuroinflammation is suspected to play an important role in the severity of anti-NMDAR encephalitis, we examined the CSF levels of NLRP3 inflammasome and relevant inflammatory cytokines in patients with anti-NMDAR encephalitis, viral meningoencephalitis and non-inflammatory CNS disease. In this study, we aimed to find out the correlation between clinical outcomes and NLRP3 inflammasome, and to investigate whether NLRP3 can be used as a diagnostic and prognostic factor for anti-NMDA receptor encephalitis in clinical practice.

## Materials and Methods

### Patients and Controls

We recruited 25 patients with anti-NMDAR encephalitis, 12 patients with viral meningoencephalitis (VM) and 26 controls with non-inflammatory neurological diseases from the Department of Neurology, Nanfang Hospital, Southern Medical University. The revised anti-NMDAR encephalitis diagnosis criteria of 2016 were used as the inclusion criteria for the anti-NMDAR encephalitis group (21). All CSF samples of anti-NMDAR encephalitis patients were positive for NR1 subunit of anti-NMDAR antibodies by cell-based analysis (5) and negative for viral DNA or other pathogens. All anti-NMDAR encephalitis patients were treated with plasmapheresis, intravenous immunoglobulin or high dose of methylprednisolone. Only one patient stayed in hospital more than 6 months, and the other 24 patients successfully discharged 1~2 month after treatment. The VM group patients were made a definite diagnosis by detecting viral DNA such as herpes simplex virus, cytomegalovirus, and varicella-zoster virus in their CSF by PCR. The controls group consisted of patients with other CNS non-inflammatory disorders, including cerebral vascular disease and abnormal movements. Both the VM and control groups were negative for NR1 subunit of anti-NMDAR antibodies in CSF. The clinic manifestations and baseline characteristics of anti-NMDAR encephalitis patients (*n* = 25), VM patients (*n* = 12), and controls (*n* = 26) were shown in Table 1. Patients with non-inflammatory neurological disease were used as controls, including 6 cases of alzheimer's disease, 12 cases of parkinson's disease and 8 cases of normal pressure hydrocephalus. The etiologies of viral meningoencephalitis including 4 cases of herpesvirus, 2 cases of Epstein Barr virus, 3 cases of varicella zoster virus, and 3 cases of unknown etiology but responsive to antiviral therapy. In the cohort of anti-NMDAR encephalitis patients, disorders of memory, behavior, and cognition (88%) and seizure (72%) were the most common clinic manifestations.

**Table 1:** The clinic manifestations and baseline characteristics of anti-NMDAR encephalitis VM and controls.

	**NMDAR (*n* = 25)**	**VM (*n* = 12)**	**Control (*n* = 26)**
Gender (male/female)	11/14	7/5	13/13
Age (years)	35.5 ± 19.7	34.5 ± 15.8	38.8 ± 16.2
Psychiatric and neurologic symptoms			
Fever	14 (56%)	8 (67%)	–
Disorders of memory, behavior, and cognition	22 (88%)	4 (33%)^***^	–
Seizures	18 (72%)	3 (25%)^**^	–
Autonomic disturbances	10 (40%)	2 (17%)	–
Disturbance of consciousness	15 (60%)	6 (50%)	–
Abnormal movements	11 (44%)	2 (17%)	–
ovarian teratoma	3 (12%)	0 (0%)	–
CSF white blood cell count ( × 10^6^/L, median)	4(1,18)	3 (0.58)	0 (0, 0)^***^
CSF protein(g/L, median)	0.30 (0.17, 0.76)	0.25 (0.18, 0.94)	0.28 (0.19, 0.40)
Maximum mRS scores	4, (4,5)	-	-
6 months' mRS scores after the disease onset	3, (2,3)	-	-
Anti-NMDAR antibody	25	0	0

** *p < 0.01*,

*** *p < 0.001*.

This study was approved by the Ethics Committee of Nanfang Hospital, Southern Medical University. Written informed consent was obtained from each participant after an explanation of the purpose and procedures of this study.

### Determination of CSF NLRP3 Inflammasomes and Other Inflammatory Cytokine Levels

After the patients were hospitalized, CSF samples were obtained immediately and centrifuged at 1,000 g for 10 min. The supernatant was then transferred into a polypropylene tubes and stored at −80°C until the assays were performed. All procedures were completed in 60 min. Commercial Sandwich Enzyme-linked immunosorbent assay (ELISA) kits were used to detect the levels of CSF NLRP3 inflammasomes (CSB-E15885h, Cusabio, Wuhan, china), IL-1β, IL-6, and IL-17 (Bender MedSystems GmbH, Vienna, Austria). The assays were performed according to the manufacturers' instructions. All standards and samples were measured in duplicate and CSF samples were measured undiluted. Optical densities were determined on a Microplate Reader (BMG LabTech).

### Clinic and Follow-Up Evaluation

The modified Rankin Scale (mRS) scores were used to evaluate the clinical neurologic disabilities. All patients with anti-NMDAR encephalitis were evaluated for mRS scores at the times of acute stage and 6 months after onset. And the former was defined as peak mRS scores, the latter was taken as the remission scores. Through follow-up, 13 out of 25 patients were re-examed for these four profiles in 3–6 months later. The other 12 patients did not come back to our hospital, or refused lumbar puncture once more. The change of NLRP3 (δNLRP3) and of mRS (δmRS) were calculated according to the following formulas: δNLRP3 = NLRP3 (acute stage) – NLRP3 (remission stage); δmRS = mRS (acute stage)—mRS (remission stage).

### Statistical Analysis

SPSS version 20.0 (IBM Corp, Armonk, NY, USA) was used for data analyzation. Data were presented as mean (±standard deviation) or median (interquartile range). Kruskal-Wallis test was used for the comparison between groups. Paired Wilcoxon tests were used to compare these profiles before and after treatment in the 13 patients who were re-evaluated during follow-up. Correlations between the profiles were evaluated using Pearson's test or Spearman's test. *p*-value < 0.05 was considered statistically significant.

## Result

### Increased Levels of NLRP3 Inflammasomes and Inflammatory Cytokines in CSF of Patients With Anti-NMDAR Encephalitis

**Figure 1:**
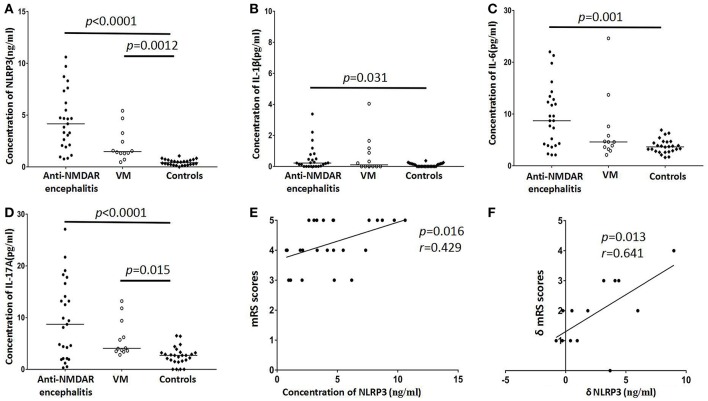
Distribution of CSF NLRP3 inflammasomes and cytokines levels in anti-NMDAR encephalitis patients and controls groups [viral meningoencephalitis (VM) and non-inflammatory neurological diseases (Controls)]. Higher levels of CSF NLRP3 inflammasomes were found in anti-NMDAR encephalitis and VM patients **(A)**. CSF IL-1β levels of anti-NMDAR encephalitis patients had significant difference with controls **(B)**. CSF levels of IL-6 showed elevated in patients with anti-NMDAR encephalitis **(C)**. The levels of IL-17 were also significantly changed in anti-NMDAR encephalitis and VM patients **(D)**. The correlation between mRS score and CSF NLRP3 inflammasomes in anti-NMDAR encephalitis patients in acute stage **(E)**. The correlation between the δmRS and the decrease of CSF NLRP3 inflammasomes in patients with anti-NMDAR encephalitis in follow-up period **(F)**. The *p*-values and *r* values were indicated within figures. (mRS, modified Rankin Scale; VM, viral meningoencephalitis).

In the present study, CSF NLRP3 inflammasomes, IL-1β, IL-6, and IL-17 were detected in anti-NMDAR encephalitis patients and two control groups and the data were shown in Table 1 and Figure 1. The CSF levels of NLRP3 inflammasomes, IL-1β, IL-6, and IL-17 inflammasome was notably increased in anti-NMDAR encephalitis patients in acute stage compared with controls (*p* < 0.001, *p* = 0.031, *p* = 0.001, *p* < 0.001, respectively). The NLRP3 inflammasomes, and IL-17 also were found to be markedly higher in VM patients than in controls (*p* = 0.0012, *p* = 0.015; respectively). In the 13 followed-up patients with anti-NMDAR encephalitis, the levels of CSF NLRP3 inflammasome, IL-1β, IL-6, and IL-17 were decreased in remission stage compared with that in acute stage (*p* = 0.012, *p* = 0.005, *p* = 0.013, *p* = 0.001, respectively).

### Relationship Between CSF NLRP3 and Inflammatory Cytokines

In each group, we detected correlation between levels of CSF NLRP3 inflammasome and the other three interleukins. In patients with anti-NMDAR encephalitis, positive correlations were found between NLRP3 and IL-1β, IL-6, or IL-17 (*p* < 0.001, *r* = 0.676; *p* < 0.001, *r* = 0.690; *p* = 0.006, *r* = 0.492, respectively). NLRP3 was also positively correlated with IL-1β and IL-6 in the VM group. No correlations were found between NLRP3 and cytokines in the control group of non-inflammatory neurological diseases.

### Relationship Between Clinical Outcomes and CSF NLRP3 or Inflammatory Cytokines in Anti-NMDAR Encephalitis

We also detected a positive correlation between peak mRS score and the level of CSF NLRP3 inflammasome in anti-NMDAR encephalitis patients (*p* = 0.016, *r* = 0.429, Figure 1E). Peak mRS score was positively correlated with IL-6 (*p* = 0.005, *r* = 0.504) and IL-17 (*p* = 0.023, *r* = 0.403). In the 13 re-examed anti-NMDAR encephalitis patients, we analyzed the correlation between the reduction of mRS score and the decrease of NLRP3 inflammasome, IL-1β, IL-6, and IL-17. There was a positive correlation between δmRS and the decrease of NLRP3 inflammasome (*p* = 0.013, *r* = 0.641, Figure 1F), but not IL-1β, IL-6, or IL-17.

## Discussion

It is still a challenge for clinicians to accurately diagnose CNS inflammatory diseases. Anti-NMDAR encephalitis is an inflammatory disease mediated by anti-neuronal antibody in the CNS. Patients presenting with characteristic progressive neuropsychiatric symptoms and non-specific evidence of CNS inflammation are usually diagnosed as anti-NMDAR encephalitis (22). Most importantly, since that early treatment contributed to a better prognosis, it's crucial to recognize anti-NMDAR encephalitis timely (23). Here, we sought to prove the diagnostic and prognostic value of NLRP3 inflammasome in anti-NMDAR encephalitis.

Some interesting studies have explored the relationship between NMDAR and NLRP3 inflammasomes, but the results are still controversial. In the murine model of NMDA-induced retinal excitotoxicity, activation of NMDAR can prime the NLRP3 inflammasome in a transcription-dependent manner (24). But in the models of acute hepatitis and pancreatitis, activation of NMDAR finally down-regulates NLRP3 inflammasomes via a β-arrestin-2 NF-kβ and JNK pathway (25). Under the stimulation of pathogen-associated molecular patterns (PAMPs) or damage-associated molecular patterns (DAMPs), NLRP3 inflammasome activates caspase-1, which subsequently leads to mutation IL-1β through the cleavage of pro-IL-1β and finally results in cascade inflammatory response (26). In the present study, we detected a significant increase of CSF NLRP3 inflammasomes and its downstream cytokines in anti-NMDAR encephalitis patients in acute stage, and a decrease in their respective levels during the remission stage. It suggested that NLRP3 inflammasomes might participate in the pathogenesis of anti-NMDAR encephalitis. In this disease, activated NLRP3 inflammasomes amplifies the inflammatory cascade response by activating downstream IL-1β and other inflammatory cytokines, resulting in brain damage, and neuropsychiatric symptoms. B cells have already been confirmed to take part in anti-NMDAR encephalitis (27). However, the mechanism concerning their entry into the CNS compartment, or the blood-brain barrier, is unclear. Some studies in other CNS autoimmune disease such as experimental autoimmune encephalomyelitis (EAE), have demonstrated that NLRP3 inflammasome promotes neuroinflammation by inducing the migration of Th1 and Th17 cell to CNS (28, 29). Mature IL-1β, which can be indirectly activated by NLRP3 inflammasome, was proved to be important in Th17 differentiation (30). Th17 cells produce IL-17 and IL-6, two pro-inflammatory cytokines that could regulate Th17/Treg (regulatory T cells) balance and were increased in antibody-mediated CNS disorders, such as neuromyelitis optical (NMO) (31, 32). Our study also found the increase of IL-6 and IL-17 in anti-NMDAR encephalitis patients, and there were positive correlations between the CSF NLRP3 inflammasome and IL-6 or IL-17. Both suggested that IL-17/IL-6 co-activation in anti-NMDAR encephalitis might be of great importance in the pathogenesis of anti-NMDAR encephalitis.

Many researches have demonstrated that the NLRP3 gene knockout animal could develop less neuroinflammation of CNS (26, 29). The NLRP3 inflammasome has been considered as the common cardinal pathology mediators in these diseases. Some drugs and chemicals such as anakinra, protein kinase A (PKA), minocycline and dimethyl sulfoxide have been proved to attenuate neuroinflammation by targeting pathways upstream and downstream of NLRP3 inflammasome signaling (33), which offer potential new therapeutics for these CNS autoimmune diseases, including anti-NMDAR encephalitis (11). Our study also revealed the increased CSF NLRP3 inflammasome as an indicator for the severity of anti-NMDAR encephalitis, so the future researches should focus on the attenuating effects of these potential therapeutical drugs in anti-NMDAR encephalitis *in vitro* or *in vivo*.

## Conclusion

In this study, we found significant increases of CSF NLRP3 inflammasome, IL-1β, IL-6, and IL-17 in anti-NMDAR encephalitis patients in acute stage compared with patients with VM as well as non-inflammatory disease controls. Moreover, in anti-NMDAR encephalitis patients, we found a positive correlation between peak mRS and CSF NLRP3 inflammasome, and the same with δmRS and the decrease of NLRP3 inflammasome. Our study suggested that the CSF levels of NLRP3 inflammasomes reflect the underlying neuroinflammatory processes in patients with anti-NMDAR encephalitis, which can act as an indicator for the severity and prognosis of anti-NMDAR encephalitis.

## Ethics Statement

The study was conducted with the approval of the Ethics Committee of Nanfang Hospital, Southern Medical University (NFEC-2018-095).

## Author Contributions

HW conceptualized the study and designed the experiments. H-YS and SuP participated in the research design. BL, DZ, ZW, TJ, H-YS, and ShP collected the CSF samples and clinical data. YP analyzed the data and wrote the manuscript.

### Conflict of Interest Statement

The authors declare that the research was conducted in the absence of any commercial or financial relationships that could be construed as a potential conflict of interest.
